# Familial amyloidotic polyneuropathy in Crete, Greece

**DOI:** 10.1186/1750-1172-10-S1-O6

**Published:** 2015-11-02

**Authors:** Minas Tzagournissakis, Cleanthe Spanaki, Georgios Amoiridis, Demetrios Samonakis, Andreas Plaitakis, Panayiotis Mitsias

**Affiliations:** 1 University Hospital of Herakion, Neurology, 71500, Heraklion, Greece; 2 University Hospital of Herakion, Gastroenterology, 71500, Heraklion, Greece

## Background

Familial amyloidotic polyneuropathy (FAP) has been related to more than 100 transthyretin (TTR) gene mutations. The disease has been reported among different ethnic groups including some kindreds of Greek origin. Here we report the clinical and molecular data, as well as treatment outcome of all Cretan patients with FAP seen at the University Hospital of Heraklion-Crete.

## Methods

Seventeen patients (8 men and 9 women), members of 6 unrelated families originating from 4 different foci on the island were studied. All had a positive family history for polyneuropathy. Extended pedigrees spanning 5 generations were constructed. All patients underwent thorough clinical and laboratory investigation including rectal and/or nerve biopsy as well as molecular analysis.

## Results

The mean age of disease onset was 30 years (range: 27 to 43). All patients presented with paresthesias, temperature loss and progressive weakness at the lower extremities, urinary difficulties, diarrhea, postural dizziness and weight loss. The upper extremities were involved later during the disease progression. Neurological examination revealed loss of pain and temperature sensation in a glove and stocking distribution and distal weakness. All but two exhibited orthostatic hypotension. Four patients presented with carpal tunnel syndrome. Although cardiac arrhythmia was a common symptom to most patients, heart failure developed in 3 patients during the late phase of the disease. One patient presented with chronic kidney disease for which she was treated with hemodialysis. Electromyographic examination revealed evidence of denervation in the muscles of the lower limbs. Conduction velocities were slightly below the normal range. Rectal and/or sural nerve biopsy revealed the presence of amyloid deposit. Molecular analysis showed that all patients were heterozygotes for the TTR Met30 mutation. Eleven patients underwent orthotopic liver transplantation (OLTx) from 1993 to 2013. Eight of them showed remarkable improvement especially of their autonomic symptoms and muscle strength. They gained weight and their paresthesias also subsided. Of the operated patients, two died of post-operative complications, one of intracerebral hemorrhage and one of unrelated cause.

## Conclusion

FAP that occurs on the island of Crete is due to Met30 mutation. Haplotype analysis that is in progress may help to elucidate the origin of this mutation in relation to other populations. Our results regarding the liver transplantation corroborate those of other groups suggesting that this is the most effective treatment currently available for FAP.

